# Supplementary material for: Association of the malate dehydrogenase-citrate synthase metabolon is modulated by intermediates of the Krebs tricarboxylic acid cycle
Source: Sci Rep. 2021 Sep 21;11:18770. doi: 10.1038/s41598-021-98314-z (PMC8455617; doi:10.1038/s41598-021-98314-z)
Supplement: Supplementary file 1 — Supplementary Information. [file 41598_2021_98314_MOESM1_ESM.pdf]

Supplementary Information

Association of the malate dehydrogenase-citrate synthase metabolon is modulated by intermediates of the Krebs tricarboxylic acid cycle

Joy Omini, Izabela Wojciechowska, Aleksandra Skirycz, Hideaki Moriyama, Toshihiro Obata

Table S1. Simulation results in ligand binding by Swiss Dock.

Ligand	Total listed binding sites	Cluster	Energy in cluster 0	Energy in cluster 9
Acetyl-CoA	256	32	−15.6943	−29.8399
α-ketoglutarate	256	44	−17.7629	−4.75648
succinyl-CoA	256	32	−35.1062	−26.976
ATP	252	34	−35.9538	−30.2348

Energy (no dimension), relative energies provided by the Swiss Dock server. The greater the negative values, the stronger the binding.

Table S2. Properties of CS A subunit 65Arg–60Ser in complex with MDH A subunit.

Parameters	Open (1cts)	Close (2cts)	Difference
ZDOCK Score (No. of selected structure)	644.695 (4)	742.992 (12)	+98.297 (+8) in 2cts
Accessible Surface Area, Å^2	284.54	325.35	+40.81 in 2cts
Buried Surface Area before binding, Å^2	0.00	0.00	0.00
Buried Surface Area after binding, Å^2	214.35	280.46	+66.11 in 2cts
Hydrogen bonds	4	6	+ 2 in 2cts
	## Structure 1 Dist. [Å] Structure 2<br>1 A:TYR 69[N] 3.51 A:ASP 88[O]<br>2 A:ARG 67[NE] 3.75 A:ASP 88[OD1]<br>3 A:ARG 65[NH2] 3.32 A:ASP 88[OD2]<br>4 A:ASP 12[OD1] 2.88 A:ASN 312[ND2]	## Structure 1 Dist. [Å] Structure 2<br>1 A:LYS 76[NZ] 2.60 A:VAL 249[O]<br>2 A:ARG 65[NH1] 2.78 A:ASN 278[O]<br>3 A:ARG 20[NH1] 2.82 A:GLU 289[OE2]<br>4 A:GLU 104[OE2] 3.55 A:ARG 167[NH2]<br>5 A:TYR 69[OH] 3.53 A:CYS 188[N]<br>6 A:THR 103[OG1] 3.71 A:VAL 192[N]	
Salt bridges	4	4	0
	## Structure 1 Dist. [Å] Structure 2<br>1 A:ARG 67[NE] 3.75 A:ASP 88[OD1]<br>2 A:ARG 67[NH2] 3.73 A:ASP 88[OD1]<br>3 A:ARG 65[NH2] 3.32 A:ASP 88[OD2]<br>4 A:LYS 16[NZ] 3.17 A:GLU 308[OE1]	## Structure 1 Dist. [Å] Structure 2<br>1 A:ARG 20[NH1] 2.82 A:GLU 289[OE2]<br>2 A:ARG 20[NH2] 3.70 A:GLU 289[OE2]<br>3 A:GLU 104[OE2] 3.55 A:ARG 167[NH2]<br>4 A:GLU 104[OE2] 3.55 A:ARG 167[NH2]	

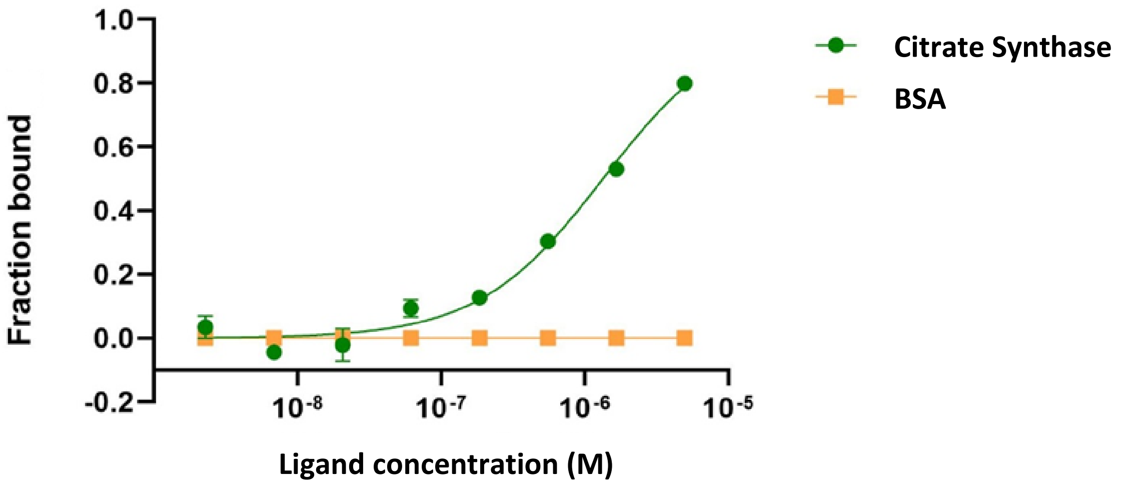

Figure S1. Microscale thermophoresis (MST) analysis of the MDH-CS multi-enzyme complex. The affinity of the MDH-CS multi-enzyme complex was analyzed by MST using fluorescently labeled MDH as the target. CS (green) and bovine serum albumin (orange) were used as the ligand proteins. Curves represent the response (fraction bound) against the concentrations (M) of ligand proteins. Error bars represent the standard deviations of three measurements.

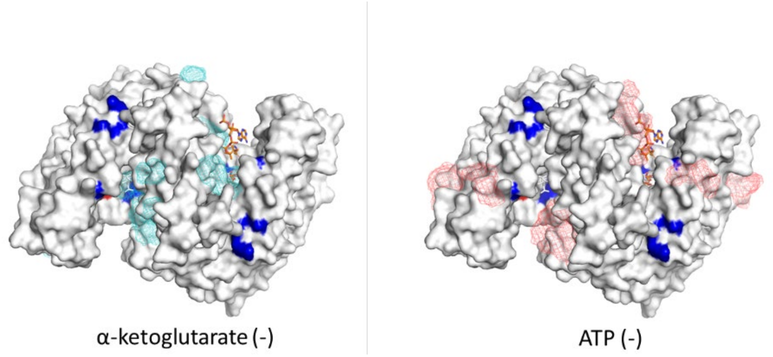

Figure S2. Predicted ligand binding sites in CS apoenzyme. The white surface models of the CS apoenzyme in the open format are shown. The 274His and 320 His residues at the reaction center and the 65Arg and 67Arg residues that are involved in the MDH-CS interaction are highlighted in blue. The mesh of blue, green, and magenta indicate the positions of α-ketoglutarate and ATP at the predicted binding sites, respectively. White and orange stick models indicate the citrate and CoA in the reported crystal structure, respectively.
